# Mechanisms of Supralinear Calcium Integration in Dendrites of Hippocampal CA1 Fast-Spiking Cells

**DOI:** 10.3389/fnsyn.2018.00047

**Published:** 2018-12-11

**Authors:** Olivier Camiré, Ivan Lazarevich, Tommy Gilbert, Lisa Topolnik

**Affiliations:** ^1^Department of Biochemistry, Microbiology and Bio-informatics, Faculty of Science and Engineering; Neuroscience Axis, CHU de Québec Research Center (CHUL), Laval University, Québec, QC, Canada; ^2^Lobachevsky State University of Nizhni Novgorod, Nizhny Novgorod, Russia

**Keywords:** Ca^2+^-permeable AMPA receptor, glutamate, excitatory current, synaptic plasticity, ryanodine receptor

## Abstract

In fast-spiking (FS), parvalbumin-expressing interneurons of the CA1 hippocampus, activation of the GluA2-lacking Ca^2+^-permeable AMPA receptors (CP-AMPARs) in basal dendrites is coupled to Ca^2+^-induced Ca^2+^-release (CICR), and can result in a supralinear summation of postsynaptic Ca^2+^-transients (post-CaTs). While this mechanism is important in controlling the direction of long-term plasticity, it is still unknown whether it can operate at all excitatory synapses converging onto FS cells or at a set of synapses receiving a particular input. Using a combination of patch-clamp recordings and two-photon Ca^2+^ imaging in acute mouse hippocampal slices with computational simulations, here we compared the generation of supralinear post-CaTs between apical and basal dendrites of FS cells. We found that, similar to basal dendrites, apical post-CaTs summated supralinearly and relied mainly on the activation of the CP-AMPARs, with a variable contribution of other Ca^2+^ sources, such as NMDA receptors, L-type voltage-gated Ca^2+^-channels and Ca^2+^ release. In addition, supralinear post-CaTs generated in apical dendrites had a slower decay time and a larger cumulative charge than those in basal, and were associated with a stronger level of somatic depolarization. The model predicted that modulation of ryanodine receptors and of the Ca^2+^ extrusion mechanisms, such as the Na^+^/Ca^2+^-exchanger and SERCA pump, had a major impact on the magnitude of supralinear post-CaTs. These data reveal that supralinear Ca^2+^ summation is a common mechanism of Ca^2+^ signaling at CP-AMPAR-containing synapses. Shaped in a location-specific manner through modulation of ryanodine receptors and Ca^2+^ extrusion mechanisms, CP-AMPAR/CICR signaling is suitable for synapse-specific bidirectional modification of incoming inputs in the absence of active dendritic conductances.

## Introduction

In cortical networks, GABAergic inhibitory interneurons play a crucial role in the development and maturation of neural circuits, regulation of synaptic plasticity and rhythmogenesis (Cossart, 2011; Lehmann et al., 2012; Allen and Monyer, 2014). These cells form a highly heterogeneous population with distinct gene expression as well as morphological, molecular, and physiological properties that fit interneuron type-specific function (Klausberger and Somogyi, 2008; Kepecs and Fishell, 2014; Paul et al., 2017; Pelkey et al., 2017). The synaptic organization of inhibitory interneurons, with distinct transmission properties and plasticity mechanisms operating at individual synapses, adds another level of complexity in understanding interneuron function. Therefore, recent efforts have been undertaken to demystify the cell- and synapse-type specific forms of postsynaptic signaling and to identify the common rules important for interneuron learning.

Among ionotropic glutamate receptors, the GluA2-lacking Ca^2+^ -permeable alpha-amino-3-hydroxi-5-methyl-4-isoxazolepropionic acid (AMPA) receptors (CP-AMPARs) provide fast postsynaptic Ca^2+^ influx that is associated with the induction of distinct forms of synaptic plasticity in interneurons (Mahanty and Sah, 1998; Laezza et al., 1999; Liu and Cull-Candy, 2000; Goldberg et al., 2003a,b; Topolnik et al., 2005; Soler-Llavina and Sabatini, 2006; Lamsa et al., 2007; Oren et al., 2009; Croce et al., 2010; Nissen et al., 2010; Szabo et al., 2012; Camiré and Topolnik, 2014; Hainmuller et al., 2014; Lalanne et al., 2016). Parvalbumin (PV)-expressing fast-spiking (FS) cells, including basket cells in neocortical (Goldberg et al., 2003a,b) and hippocampal (Camiré and Topolnik, 2014; Hainmuller et al., 2014) regions and cerebellar stellate cells (Soler-Llavina and Sabatini, 2006) are particularly enriched with CP-AMPARs, which play a critical role in dendritic Ca^2+^ compartmentalization in the absence of dendritic spines. Specifically, the interaction between CP-AMPARs, the Ca^2+^ binding protein PV, and the membrane Na^+^/ Ca^2+^ exchanger (NCX) have been involved in the formation of highly localized dendritic Ca^2+^ microdomains during basal synaptic activity (Goldberg et al., 2003a; Soler-Llavina and Sabatini, 2006). The inward rectification of the CP-AMPAR-mediated current due to increased blockade by endogenous polyamines at depolarized levels of membrane potential (Koh et al., 1995) is associated with a strong attenuation of the CP-AMPAR Ca^2+^ influx (Topolnik et al., 2005) and dictates the rules for the induction of synaptic plasticity at excitatory inputs to interneurons (Lamsa et al., 2007). Indeed, CP-AMPAR-mediated Ca^2+^ influx is involved in the induction of anti-Hebbian long-term potentiation (LTP) under basal levels of synaptic activity in interneurons at rest (Camiré and Topolnik, 2014). Increasing activity levels during synchronous theta-bursting of CA1 pyramidal cells leads to additional activation of Ca^2+^ -induced Ca^2+^ release (CICR) via ryanodine receptors (RyRs) and generation of supralinear Ca^2+^ signals in dendritic microdomains (Camiré and Topolnik, 2014). As a result, this CP-AMPAR-triggered CICR is involved in the induction of long-term depression (LTD) (Camiré and Topolnik, 2014). Thus, CP-AMPARs are able to play a dual role as the triggering factor in both LTP and LTD induction, which indeed was observed in different cell types and synapses (Nissen et al., 2010; Hainmuller et al., 2014).

Interestingly, the hippocampal CA1 *oriens/alveus* (O/A) FS cells, including basket and bistratified cells, switch from LTP to LTD following transition from small to supralinear Ca^2+^ signals via CP-AMPARs in basal dendrites. Whether the apical dendrites of these cells may demonstrate supralinear Ca^2+^ signals triggered upon CP-AMPAR-dependent CICR remains unknown. To understand the input-specific organization of dendritic Ca^2+^ signaling in FS interneurons, here we compared the summation of postsynaptic Ca^2+^ transients (post-CaTs) between apical and basal dendrites, and further explored the common mechanisms that may shape the supralinear post-CaTs in dendritic compartments of FS cells. Our data indicate that CP-AMPAR-mediated Ca^2+^ influx is a common mechanism operating in FS interneuron dendrites, regardless of the input-specific innervation patterns. We also predict that the subcellular distribution and functional state of Ca^2+^ sources and Ca^2+^ extrusion mechanisms will have a major impact on supralinear Ca^2+^ dynamics and, accordingly, on the direction of synaptic plasticity in CP-AMPAR-expressing interneurons.

## Materials and Methods

### Mice

All experiments were conducted in accordance with the Canadian Council on Animal Care in Science and with approval of the Laval University Animal Protection Committee. CD1 mice (P14–22; *n* = 26) were used in this study. This age range is prior to the onset of puberty, and the results obtained from both sexes were combined.

### Electrophysiological Recordings

Animals were anesthetized with isoflurane and decapitated. The brain was rapidly removed into ice-cold, oxygenated “cutting” solution containing (in mM) 220 or 250 sucrose, 2 KCl, 1.25 NaH_2_PO_4_, 26 NaHCO_3_, 7 MgSO_4_, 0.5 CaCl_2_, and 10 glucose (pH = 7.4, 320–340 mOsm). Transverse hippocampal slices (300 μm thick) were cut with a Vibratome (Microm, Fischer Scientific, USA), transferred to heated (35°C) oxygenated recovery solution containing (mM) 124 NaCl, 2.5 KCl, 1.25 NaH_2_PO_4_, 26 NaHCO_3_, 3 MgSO_4_, 1 CaCl_2_, and 10 glucose (pH = 7.4, 300 mOsm), and allowed to cool down to room temperature in 30 min. Slices were allowed to recover for at least 1 h before experiments.

**Figure 1:**
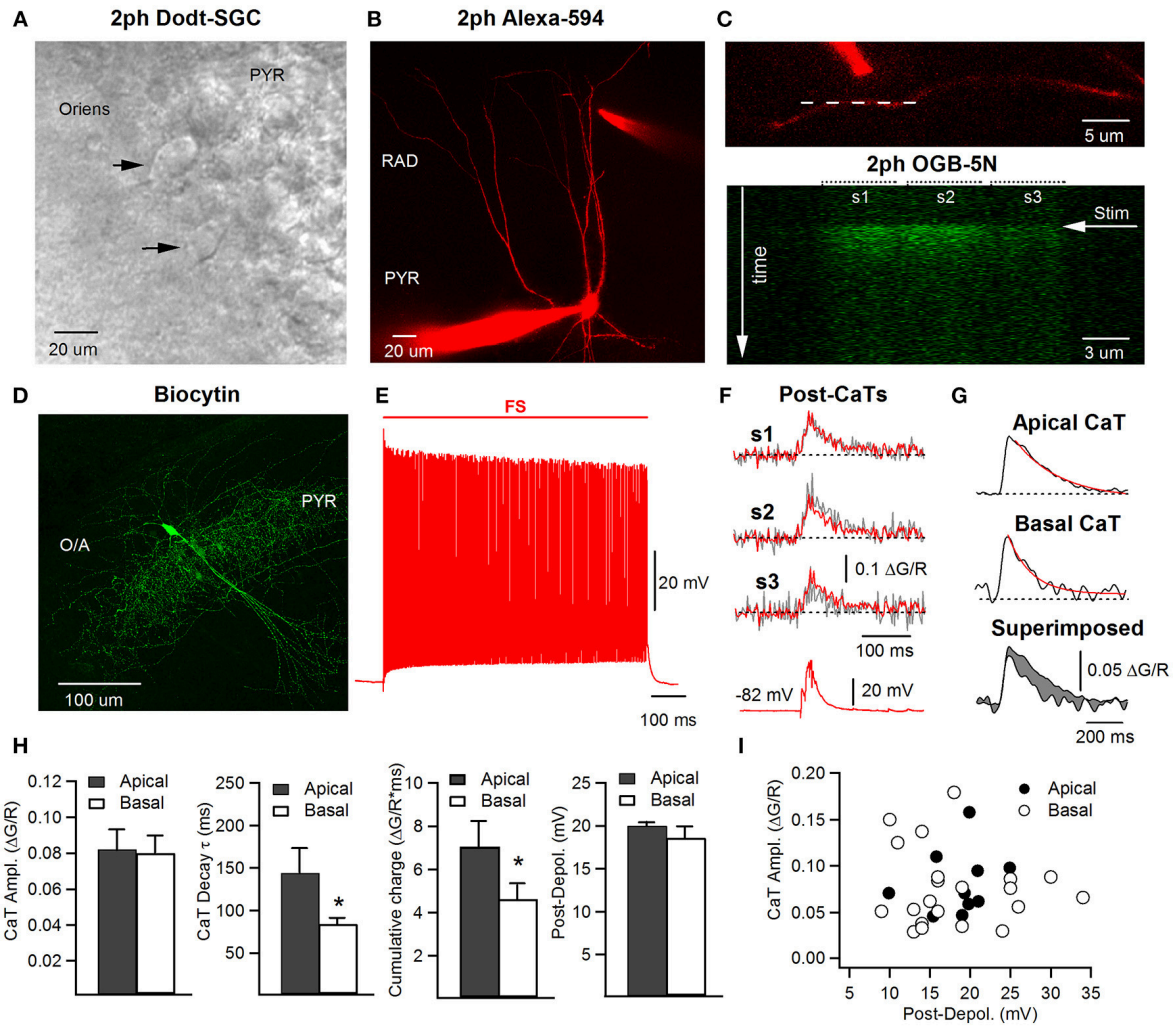
Two-photon imaging of dendritic Ca^2+^ transients in hippocampal CA1 FS cells. **(A)** Two-photon Dodt scanning gradient contrast (2ph Dodt-SGC) image showing a location of putative FS interneurons (black arrowheads) in O/A close to pyramidal layer. **(B)** Two-photon image (maximal projection of a Z-stack) of an interneuron filled during recording with a morphological dye (Alexa-594) and a Ca^2+^-sensitive indicator (OGB-5N), with a stimulation theta-glass bipolar electrode filled with ACSF containing Alexa-594 and positioned next to the apical dendrite. **(C)** Zoomed in two-photon image showing a dendrite of interest with the stimulation electrode positioned next to it, and the position of the line scan along the dendrite indicated with a white dotted line (upper); the lower image corresponds to a single line scan acquisition of the OGB-5N fluorescence signal in three neighboring dendritic segments (s1 to s3) in response to electrical stimulation indicated with a white horizontal arrow. **(D)** Confocal image (maximal projection of a Z-stack) of an interneuron filled during recording with biocytin and identified as a basket cell. **(E)** A typical firing pattern of an FS interneuron in response to somatic current injection (0.8–1 nA, 1 s). **(F)** Representative postsynaptic Ca^2+^ transients (post-CaTs; individual trace is shown in gray and average trace obtained by averaging 4 individual traces is shown in red) evoked in dendritic segments illustrated in **(C)** in response to a burst of 3 stimuli applied to the cell held in current-clamp at −82 mV (lower trace). **(G)** Representative Ca^2+^ traces (averaged) in apical (top) and basal (middle) dendrites, with both superimposed (bottom), showing a slower apical Ca^2+^ decay. Red trace represents the fitted exponential decay curve. The difference in cumulative charge (area under the curve) is shown in gray (bottom). **(H)** Summary bar graphs of the post-CaT peak amplitude (left), of the CaT decay time constant and area under the curve (cumulative charge; middle) and of the peak amplitude of the corresponding postsynaptic depolarization (Post-Depol; right) in apical (gray) and basal (white) dendrites. **(I)** Summary plot showing the relationship between the CaT peak amplitude and the level of postsynaptic depolarization in apical (black) and basal (white) dendrites. No correlation was revealed between the amplitude of post-CaTs and the postsynaptic depolarization, in line with soma-independent Ca^2+^-signaling in FS interneuron dendrite. *p < 0.05.

During experiments, slices were perfused continuously (2.5 ml/min) with artificial cerebro-spinal fluid (ACSF) at 30–33°C. CA1 interneurons of *stratum oriens/alveus* were identified with the aid of an infrared-scanning Dodt gradient contrast (Figure 1A) or an infrared camera (70 Series, DAGE-MTI, Michigan, USA) mounted on an upright microscope (Leica DM6000, Leica Microsystems Inc., Mississauga, ON, Canada) equipped with a long-range water-immersion objective (40x, 08NA). Whole-cell current-clamp recordings were made from somata using a Multiclamp 700B amplifier (Molecular Devices, Union City, CA, USA). Recording pipettes (3.5-5 MΩ) were filled with a solution containing (in mM) 130 KMeSO_3_, 2 MgCl_2_, 10 diNa-phosphocreatine, 10 HEPES, 2 ATPTris, 0.2 GTPTris, 0.02 Alexa Fluor-594 (Life Technologies, Carlsbad, CA, USA), 0.5 Oregon green-488-BAPTA-5N-hexapotassium salt (OGB-5N, Life Technologies, Carlsbad, CA, USA), and 0.2–0.3% biocytin (pH 7.25–7.35, 275–290 mOsm). Current clamp recordings were performed at −65 to −85 mV. Data were low-pass filtered at 2 KHz, digitized at 10 KHz and stored on a computer using a data acquisition board (Digidata1400A, Molecular Devices, Union City, CA, USA) and Clampex 10.3 software (Molecular Devices, Union City, CA, USA).

Local synaptic stimulation in the *stratum radiatum* (RAD) or *oriens/alveus* (O/A) was achieved using a bipolar stimulating electrode made from the borosilicate theta-glass capillaries (BT-150-10, Sutter Instruments, Novato, CA), filled with ACSF containing 5–10 μM Alexa Fluor 594 and positioned ~10 μm from the dye-filled dendrite. The electrode was connected to a constant current isolation unit (A360LA; World Precision Instruments Inc., Sarasota, FL, USA). Synaptic responses were evoked by a burst of 3 stimuli (25–100 μA, 0.1 ms, 100-Hz).

### Two-Photon Ca^2+^-Imaging of Interneuron Dendrites

After obtaining the whole-cell configuration, 20–30 min were allowed for intracellular diffusion of the fluorescent dye. Imaging was performed using a two-photon laser scanning microscope (Leica SP5, Leica Microsystems Inc., Mississauga, ON, Canada) with a mode-locked Ti:Sapphire laser (Chameleon Ultra II, Coherent, Missisauge, ON, Canada) operated at 800 nm wavelength, 80 MHz pulse repeat, 140 fs pulse width. A long-range water-immersion objective (40 ×, numerical aperture 0.8) was used. Fluorescence was detected through external photomultiplier tubes, and images were acquired using the Leica LAS software (Leica Microsystems Inc., Mississauga, ON, Canada). Fluorescence signals were collected by scanning a line along the dendrite of interest (total length: ~5–20 μm). Fluorescence transients were measured at 50–200 μm from the soma. The image focus of the line was carefully checked and occasionally adjusted for possible drift.

### Pharmacology

Postsynaptic Ca^2+^-transients and associated voltage responses were examined in presence of GABA_A_ and GABA_B_ receptor antagonists gabazine (1 μM) and CGP55845 (2 μM), respectively. In some experiments, the NMDA receptor antagonist DL-AP5 (100 μM), the SERCA antagonist CPA (30 μM) and the L-type VGCC blocker nifedipine (10 μM), or nimodipine (10 μM) were added to the extracellular solution. All compounds were prepared as stock solutions, frozen at −20°C, and diluted on the day of experiment.

### Data Analysis

Ca^2+^ measurements and electrophysiological recordings were analyzed using the Leica LAS software, Clampfit 10.3 and Igor Pro (Wavemetrics, Lake Oswego, OR, USA). Fluorescence changes were quantified as increases in green OGB-5N fluorescence from the baseline normalized to the red Alexa-594 fluorescence: ΔG/R = (G − G_0_)/R or normalized to the baseline green fluorescence: ΔF/F = (F − F_0_)/F_0_. Data from dendritic branches was binned and analyzed into segments of 5 μm. Original raw traces from fluorescence experiments were filtered with a Gaussian filter and transformed to changes in Ca^2+^ concentration using the expression (Maravall et al., 2000):

(1) $$\begin{array}{r} \frac{\Delta \left[Ca2+\right]}{K_{D}}=\frac{f_{\max}}{f_{0}}\left(1-R_{f}^{-1}\right)\frac{\delta f_{\max}}{\left(\delta f_{\max}-\delta f\right)\delta f_{\max}} \end{array}$$

where δ*f* is the relative fluorescence change (*F*/ *F*), *K*_*D*_ is the affinity of the Ca^2+^ indicator (25.8 μM for the OGB-5N dye), *f*_max_ is the maximum fluorescence, *R*_*f*_ = *f*_max_/ *f*_min_ is the dynamic range of the dye (*R*_*f*_ = 33.77 for 500 μM of OGB-5N; Stocca et al., 2008). The maximum relative fluorescence change was determined experimentally or using an expression

(2) $$\begin{array}{r} \delta f_{\max}=\frac{1-R_{f}^{-1}}{R_{f}^{-1}+\;{\left[Ca2+\right]}_{rest}/K_{D}} \end{array}$$

The resting Ca^2+^concentration was taken to be [*Ca*2+]_*rest*_ = 71 nM (Aponte et al., 2008). The experimentally measured values Δ*G*/*R* are linearly related to δ*f* (Δ*F*/*F*):

(3) $$\begin{array}{r} \delta f=\frac{R}{G_{0}}\frac{\Delta G}{R} \end{array}$$

where *R* and *G*_0_ are the baseline levels of red and green fluorescence, respectively. These values were determined experimentally. Decay kinetics of Ca^2+^ transients were fitted using single exponential fitting algorithms of Igor Pro.

### Computational Model of the Cell

To simulate the electrical activity of a PV+ FS cell, we used a detailed compartmental model based on the morphological reconstruction of PV+ cells (Gulyás et al., 1999). Passive parameters were distributed non-uniformly in accordance with previous assumptions (Nörenberg et al., 2010) for dentate gyrus BCs: dendritic membrane resistance was changing linearly with distance from the soma from proximal *R*_*m, prox*_ = 8 *kΩ* cm^2^ to distal *R*_*m, dist*_ = 70 *kΩ* cm^2^. The membrane capacitance *C*_*m*_ = 0.93 μF cm^−2^ and axial resistance *R*_*i*_ = 172 Ωcm were uniform throughout the cell. The reversal potential of the leak current was set to −65 mV. Model neuron's dendrites also contained uniformly distributed delayed-rectifier type K^+^ channels with a maximal conductance of 250 *S*/*m*^2^. Soma contained transient Na^+^, delayed-rectifier type K^+^ and leak channels with maximal conductances $g_{Na}=2,000\frac{S}{m^{2}},\;g_{K}=2,000\frac{S}{m^{2}},\;g_{leak}=\;0.1\frac{S}{m^{2}}$ (soma was meant to capture the contribution of the axonal initial segment, which was not modeled explicitly). The morphological reconstruction data was transferred to the NEURON simulation environment (Carnevale and Hines, 2006), in which all the simulations of the compartmental model were done.

### Model of Synaptic Currents

The synaptic current through CP-AMPA receptors can be modeled by a Goldman-Hodgkin-Katz (GHK) equation

(4) $$\begin{array}{r} I_{AMPA}^{j}=\frac{p_{j}z_{j}^{2}VF^{2}}{RT}\frac{\left(n_{int}^{j}-n_{ext}^{j}\exp\left(-\frac{z_{j}FV}{RT}\right)\right)}{1-\exp\left(-\frac{z_{j}FV}{RT}\right)}N_{syn}N_{AMPA} \end{array}$$

where *j* = *Ca, Na, K*, *N*_*AMPA*_ is the number of open AMPA receptors, $p_{j},z_{j},n_{int}^{j},\;n_{ext}^{j}$ are permeabilities, valences, intracellular, and extracellular concentrations of j-th ion, respectively. We set permeability ratios to *p*_*Ca*_/*p*_*Na*_ = 1.59 (Geiger et al., 1995) and *p*_*Ca*_/*p*_*K*_ = 1.79 (Koh et al., 1995). The I-V relationship of the CP-AMPAR current predicted by the GHK equation is approximately linear. However, the CP-AMPAR current exhibits a strong inward rectification. We modeled this effect by multiplying the current by the Heaviside function θ(−*V*). Considering that both the full current through CP-AMPARs and its Ca^2+^-carried fraction are negligible for voltages higher than 0 mV and are quasilinear functions of V for voltages lower than 0 mV, we reduced the model of the CP-AMPAR current to a Hodgkin-Huxley formalism:

(5) $$\begin{array}{rcl} I_{AMPA}^{full} & = & g_{AMPA}\left(t\right)\theta \left(-V\right)\left(V-E_{AMPA}\right) \end{array}$$

(6) $$\begin{array}{rcl} I_{AMPA}^{Ca2+} & = & \varepsilon I_{AMPA}^{full} \end{array}$$

where ε is a proportionality coefficient calculated from the GHK theory: ε = 0.077 (7.7%) and consistent with experimental findings of 4–12% (Burnashev et al., 1995).

CP-AMPAR containing synapse activation was modeled as a conductance change with a biexponential time course (rise time 0.5 ms, decay time 3 ms, peak synaptic conductance 2–8 nS). To convert synaptic current into Ca^2+^ concentration change rate, the number of activated synapses is multiplied by a scaling coefficient

(7) $$\begin{array}{r} \alpha =\frac{g_{syn}|V|p}{4F\pi Lr^{2}} \end{array}$$

where *g*_*syn*_ is the peak synaptic conductance, p is the fraction of CP-AMPAR current carried by Ca^2+^ ions, L is the effective length of the dendritic domain in which Ca^2+^ concentration change takes place, r is the dendritic radius. Such a strong dependence of CP-AMPAR mediated Ca^2+^ signals on dendritic membrane potential was observed experimentally in cortical FS cells (Goldberg et al., 2003b). The membrane voltage dependence on time due to synaptic activation was computed from the electrophysiological model of the cell, which is effectively uncoupled from the intracellular Ca^2+^ dynamics. The current through activation of the NMDA receptors was modeled within the Hodgkin-Huxley formalism as described elsewhere (Dayan and Abbott, 2001).

### Computational Model of an Imaging Experiment

We have tested the validity of using the Equation (1) to compute the Ca^2+^ concentration by modeling the kinetic scheme of Ca^2+^ binding to the indicator dye in the imaging experiment. Previous modeling studies (Goldberg et al., 2003a) have shown that the real spatial spread of Ca^2+^ in FS cell dendrites is overestimated by imaging with fluo-4 dye. We therefore performed a set of similar simulations for the OGB-5N dye, which was used in our experiments. In these simulations, the Ca^2+^ concentration *c* obeyed the equation

(8) $$\begin{array}{r} \frac{\;\partial c}{\partial t}=D_{c}\frac{\partial^{2}c}{\partial x^{2}}+B_{f}\left(x,t\right)+B_{m}\left(x,t\right)+\frac{c_{rest}-c}{\tau_{c}}+I_{syn} \end{array}$$

where $D_{c}=225\;\mu {\text{m}}^{2}/s$ is the diffusion coefficient of the free Ca^2+^, *x* is the coordinate along the dendrite (due to the thinness and lack of spines in FS cell dendrites 3D diffusion is reduced to a one-dimensional process); the fourth term represents exponential Ca^2+^ decay and accounts for the actions of the Na^+^- Ca^2+^ exchangers and Ca^2+^ membrane pumps; the last term represents synaptic input. The second term *B*_*m*_(*x, t*) is the flux due to Ca^2+^ binding to the dye (mobile Ca^2+^ buffer):

(9) $$\begin{array}{r} B_{m}\left(x,t\right)=-k_{on}^{m}c\left[MB\right]+k_{off}^{m}\left[CaMB\right] \end{array}$$

where the Ca^2+^-bound dye concentration [CaMB] obeys

(10) $$\begin{array}{r} \frac{\;\partial \left[CaMB\right]}{\partial t}=-B_{m}\left(x,t\right)+D_{m}\frac{\partial^{2}\left[CaMB\right]}{\partial x^{2}} \end{array}$$

The OGB-5N properties are as follows: total dye concentration [*MB*]_*tot*_ = 500 μM, diffusion coefficient of the dye $D_{m}=140\frac{\mu {\text{m}}^{2}}{s},\;k_{on}^{m}=17^{*}10^{7}{\text{M}}^{-1}s^{-1},\;K_{D}=25.8\;\mu \text{M}$ (DiGregorio et al., 1999). Description of the binding with fixed buffers was similar to Goldberg et al. (2003a). From these more detailed simulations we found that indeed the linear relationship between the Ca^2+^-bound dye concentration and the Ca^2+^ concentration holds and, thus, fluorescence measurements with OGB-5N do not significantly overestimate the spatial extent of the evoked Ca^2+^ events.

### Ca^2+^ Dynamics Model

Our Ca^2+^ dynamics model uses mathematical description of Ca^2+^ signaling mechanisms in FS cells identified previously (Camiré and Topolnik, 2014). Ca^2+^ non-linearities were mediated via activation of ryanodine receptors (RyRs). Most models of the Ca^2+^-induced Ca^2+^ release (CICR) are based on the flux through IP3 receptors (Li and Rinzel, 1994). As PV+ cells in the CA1 hippocampus may lack IP3 receptors (Hertle and Yeckel, 2007), we employed a RyR model previously developed for cardiac and epithelial cells (Keizer and Levine, 1996; Sneyd et al., 2003). The receptor is assumed to have 2 open and 2 closed states, and the RyR open-state probability was shown to be equal to

(11) $$\begin{array}{r} P_{RyR}=\frac{w\left(1+{\left(\frac{c}{K_{b}}\right)}^{3}\right)}{1+{\left(\frac{K_{a}}{c}\right)}^{4}+{\left(\frac{c}{K_{b}}\right)}^{3}} \end{array}$$

where *c* denotes Ca^2+^ concentration, and *w* is a gating variable that evolves according to a first-order kinetic equation

(12) $$\begin{array}{r} \frac{\;\partial w}{\partial t}=\frac{w_{\infty }\left(c\right)-w}{w_{\infty }\left(c\right)/k_{c}^{-}} \end{array}$$

where the steady-state activation function *w*_∞_ is

(13) $$\begin{array}{r} w_{\infty }\left(c\right)=\frac{1+{\left(\frac{K_{a}}{c}\right)}^{4}+{\left(\frac{c}{K_{b}}\right)}^{3}}{1+\frac{1}{K_{c}}+{\left(\frac{K_{a}}{c}\right)}^{4}+{\left(\frac{c}{K_{b}}\right)}^{3}} \end{array}$$

The RyR parameters are taken the same as for cardiac cells (Keizer and Levine, 1996): $k_{a}^{+}\;=$ 1,500 μM^4^*s*^−1^, $k_{a}^{-}$ = 28.8 *s*^−1^, $k_{b}^{+}=$ 1,500 μM^3^*s*^−1^, $k_{b}^{-}$ = 385.9 *s*^−1^, $k_{c}^{+}$ = 1.75 *s*^−1^, $k_{c}^{-}$ = 0.1 *s*^−1^; ${{K_{a}^{4}=\;k}_{a}^{-}/k}_{a}^{+};K_{b}^{3}=k_{b}^{-}/k_{b}^{+};K_{c}=k_{c}^{-}/k_{c}^{+}$.

To further simplify the RyR model we took into account the fact that dynamics of *w* is slow so that RyR open-state probability can be considered a stationary function of *c* on the duration of the Ca^2+^ non-linearity. We demonstrated validity of this assumption by comparing responses of the original and the reduced model to Ca^2+^ pulses of 300 ms duration and 1.2 μM amplitude. Observed differences between the steady-state approximation and the original model did not exceed 5%. We then approximated the open-state probability with a Hill function of the form

(14) $$\begin{array}{r} P_{RyR}\sim \frac{c^{n}}{c^{n}+K_{RyR}^{n}} \end{array}$$

where *n* = 4, *K*_*RyR*_ = 372 nM are the values found by least-squares fitting. The fact that the CICR-mediated flux depends only on the Ca^2+^ concentration makes the cell able to respond to synaptic inputs with a Ca^2+^ non-linearity regardless of mGluR activation (which is necessary in the case of IP3R-mediated release). We modeled the effects of caffeine (RyR activator) and ryanodine (RyR inhibitor) by changing *K*_*RyR*_ in a linear fashion:

(15) $$\begin{array}{r} K_{RyR}=K_{RyR}^{0}+\alpha_{r}\left[ryan\right]-\alpha_{c}\left[caff\right] \end{array}$$

where [ryan] and [caff] denote ryanodine and caffeine concentrations, respectively. Values of α_*c*_, α_*r*_ are chosen to reproduce experimental findings.

An inward Ca^2+^ flux to the endoplasmic reticulum (ER) created by SERCA pumps is modeled with a second-order Hill function:

(16) $$\begin{array}{r} I_{SERCA}\left(c\right)=\frac{V_{s}c^{2}}{c^{2}+K_{s}^{2}} \end{array}$$

Initial guesses for pumping parameters were set to *V*_*s*_ = 50 nM ms^−1^, *K*_*s*_ = 900 nM. The resulting equation for the cytosolic Ca^2+^ concentration *c* reads

(17) $$\begin{array}{r} \frac{\partial c}{\partial t}=\frac{\alpha c^{n}}{c^{n}+K_{RyR}^{n}}\left(c_{e}-c\right)-I_{SERCA}\left(c\right)+\frac{c_{rest}-c}{\tau_{c}}+I_{syn} \end{array}$$

where the last term *I*_*syn*_ represents Ca^2+^ influx through synaptic receptors, the linear relaxation term approximates fluxes through membrane Ca^2+^ pumps, Na^+^-Ca^2+^ exchangers and binding reactions with the indicator dye (hence τ_*c*_ is the effective intracellular Ca^2+^ decay time), *c*_*e*_ is Ca^2+^ concentration in the ER. In some simulations, the effect of Na^+^-Ca^2+^ exchangers was modeled separately with a more detailed voltage-dependent expression (Gall et al., 1999). The equation for ER Ca^2+^ concentration reads

(18) $$\begin{array}{r} \frac{1}{\gamma }\frac{\partial c_{e}}{\partial t}=-\frac{\alpha c^{n}}{c^{n}+K_{RyR}^{n}}\left(c_{e}-c\right)+I_{SERCA}\left(c\right) \end{array}$$

where γ is the ratio of the cytoplasmic volume to the ER volume. We took the value γ = 12.5 which corresponds to ER occupation of 8% of the dendritic volume (Spacek and Harris, 1997). The equations can be rewritten for the full intracellular Ca^2+^ concentration *c*_*f*_ = *c*+*c*_*e*_/γ:

(19) $$\begin{array}{r} \frac{\partial c_{f}}{\partial t}=\frac{c_{rest}-c}{\tau_{c}}+I_{syn} \end{array}$$

The equations for *c* and *c*_*f*_ comprise the Ca^2+^ dynamics model. Listed parameter values were used as initial guesses for parameter optimization which was done using downhill simplex method with the cost function being the squared difference between the model trajectory and filtered experimental Ca^2+^ trace. The Ca^2+^ dynamics system with fitted parameters was then studied on the phase plane. Numerical simulations of the model were implemented in Python programming language.

### Morphological and Neurochemical Analysis

Slices with recorded cells filled with biocytin were fixed overnight with 4% paraformaldehyde at 4°C. To reveal biocytin, slices were permeabilized with 0.5% Triton X-100 and incubated with streptavidin-conjugated Alexa Fluor 488 (1:200; Jackson ImmunoResearch, Baltimore Pike, PA) overnight at 4°C. Sections were mounted in Dako fluorescence medium (Dako Canada Inc., Mississauga, Ontario, Canada) and confocal *Z* stacks (1-μm step) of biocytin-filled interneurons were acquired using a Leica SP5 imaging system equipped with a 488 nm Argon laser. The morphological identification of recorded interneurons was achieved by the analysis of their axonal arborization.

### Statistical Analysis

All data are presented as means ± SEM. The *n* represents the number of cells studied (several dendritic segments of 5 μm each from one dendrite were analyzed per cell). As the effect of pharmacological agents could differ between neighboring dendritic segments, the total number of analyzed dendritic segments per cell is indicated for pharmacological experiments in addition to the number of cells recorded. Statistical significance was evaluated with Student's paired or unpaired *t*-test for data that presented a normal distribution, and with the Mann–Whitney test (unpaired) or Wilcoxon signed-rank test (paired) for other data. Normality was tested using the Shapiro-Wilk test (^*^p < 0.05, ^**^p < 0.01, ^***^p < 0.001).

## Results

To investigate the postsynaptic Ca^2+^ dynamics along the dendritic tree of hippocampal CA1 FS interneurons, we performed whole-cell current clamp recordings in combination with two-photon dendritic Ca^2+^-imaging (Figure 1). Putative FS interneurons were selected for patch-clamp recordings using Dodt infrared-scanning gradient contrast as cells that had large-size round somata adjacent to the CA1 pyramidal layer (Figure 1A). Only cells that exhibited FS firing pattern (180–200 Hz at 32°C; Figure 1E) were included in this study. During recordings, FS cells were filled with biocytin to allow their unambiguous morphological identification. Most recorded cells showed axonal arborizations restricted to the pyramidal layer (Figure 1D) or to the RAD and O/A, suggesting that they belong to populations of basket and bistratified cells, respectively.

To compare the summation of postsynaptic Ca^2+^-transients (post-CaTs) in dendritic sub-regions receiving anatomically and functionally distinct excitatory inputs (Takács et al., 2012; Lee et al., 2014), we examined the dynamics of post-CaTs in dendritic segments within *str. radiatum* (apical) vs. those within *str. oriens/alveus* (basal). Dendritic excitatory synapses were activated locally using bipolar stimulation through a theta-glass microelectrode positioned near the dendrite of interest (Figures 1B,C, top). Recordings were performed in current-clamp at −70 ± 5.0 mV in the presence of the GABA_A_ and GABA_B_ receptor antagonists gabazine and CGP55845, respectively. Postsynaptic CaTs (post-CaTs) evoked by minimal electrical stimulation in apical and basal dendrites had similar amplitude, but the signal's area under the curve (cumulative charge) was higher in apical dendrites, consistent with a slower, longer-lasting CaT than in basal dendrites (Figures 1F,G,H). The postsynaptic depolarization associated with CaTs was of the same amplitude regardless of whether apical or basal dendrites were stimulated (Figures 1H,I). In addition, we found no correlation between the peak amplitude of post-CaTs and that of postsynaptic depolarization for both inputs (Figure 1I), consistent with the local nature of post-CaTs generated in distal dendrites of FS cells.

**Figure 2:**
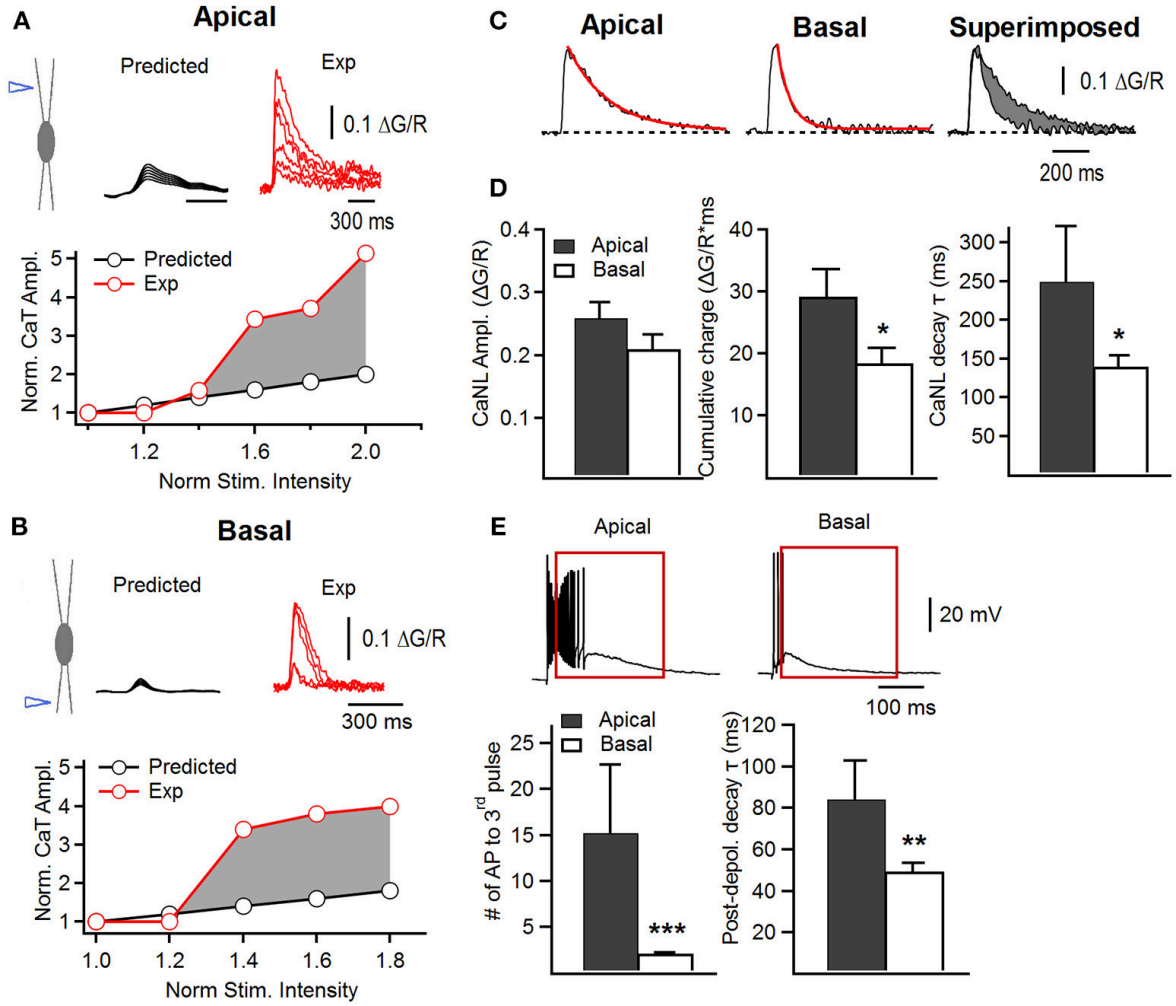
Supralinear summation of postsynaptic Ca^2+^ transients in apical and basal dendrites of FS cells. **(A,B)** Upper, schematics of electrical stimulation in the area of apical **(A)** or basal **(B)** dendrites (left) and examples of Ca^2+^ transients predicted from the linear summation of inputs (black traces) and recorded experimentally (red traces); lower, a representative plot showing the relationship between the peak CaT amplitude and stimulation intensity normalized to the value at which the smallest CaT was recorded. The data predicted from the linear relationship (black) and obtained experimentally (red) are shown superimposed with a shaded area illustrating the supralinear character of summation under experimental conditions. **(C)** Representative supralinear Ca^2+^ traces in apical (left) and basal (middle) dendrites, with both superimposed (right), showing a slower apical Ca^2+^ decay. Red trace represents the fitted exponential decay curve. The difference in cumulative charge (area under the curve) is shown in gray (right). **(D)** Summary bar graphs for the Ca^2+^ non-linearity (CaNL) peak amplitude (left), cumulative charge (middle) and decay time constant (right) in apical (black) and basal (white) dendrites. **(E)** Representative traces of the voltage changes associated with CaNLs (upper) and summary bar graphs showing the number of spikes generated in response to the third stimulus when a burst of three stimuli was applied (lower left) and the decay time constant of the postsynaptic depolarization evoked by a burst of three stimuli (lower right) in apical (black) and basal (white) dendrites. *p < 0.05; **p < 0.01; ***p < 0.001.

To compare the rules of summation of post-CaTs between apical and basal dendrites, we gradually raised the stimulation intensity up to 200% of minimal stimulation. For both inputs, the recordings were obtained in distal dendrites at similar distance to the soma (apical, distance from soma: 139.8 ± 13.1 μm, *n* = 10; basal, distance from soma: 112.5 ± 5.3 μm, *n* = 22), and in dendrites having a similar diameter (apical, 1.33 ± 0.14 μm, *n* = 9; basal, 1.24 ± 0.07, *n* = 19; *P* = 0.253). In line with previous findings in basal dendrites (Camiré and Topolnik, 2014), increasing the stimulation intensity in apical dendrites resulted in an all-or-none supralinear Ca^2+^-response (Figure 2A), indicating that supralinear Ca^2+^ integration is a common property of FS cell dendrites, regardless of the input activated. The Ca^2+^ threshold for the generation of supralinearity was similar in both apical and basal dendrites (apical ΔG/R, 0.082 ± 0.011; basal ΔG/R, 0.080 ± 0.009, *P* = 0.449). Supralinear post-CaTs also had similar amplitude in apical and basal dendrites (apical ΔG/R: 0.26 ± 0.03, *n* = 10; basal ΔG/R: 0.21 ± 0.02, *n* = 22; *P* = 0.0703; Figures 2A–D). The CaNL area under the curve, however, was significantly higher in apical dendrites than in basal ones (apical, 29 ± 4 ΔG/R^*^ms, basal, 19 ± 3 ΔG/R^*^ms, *P* = 0.0264; Figures 2C,D, middle), similar to the data obtained for the minimally evoked post-CaTs (Figure 1H). In line with this finding, the decay time constant of supralinear post-CaTs, was also significantly slower in apical dendrites (apical, 249 ± 76 ms, basal, 144 ± 16 ms, *P* = 0.034; Figures 2C,D, right). Importantly, whilst the degree of supralinearity during summation of post-CaTs was similar between the two dendritic regions resulting in a 3- to 4-fold increase in Ca^2+^-concentration when stimulation intensity was raised by 50% from the minimal level (Figures 2A,B), the overall gain in dendritic Ca^2+^-signal showed a strong degree of variability across the dendritic tree (apical, range: 3.7–11.6, variance: 7.9, *n* = 10; basal, range: 4.6–16.8, variance: 14.8, *n* = 21), indicating that dendritic site-specific mechanisms may shape the summation of post-CaTs in individual postsynaptic microdomains.

Furthermore, the supralinear post-CaTs in apical dendrites were associated with a significantly larger number of spikes (AP number to the 3rd stimulus, apical: 15.3 ± 7.3, *n* = 9; basal: 2.1 ± 0.3, *n* = 22; *P* = 0.0016, Mann-Whitney test; Figure 2E) and a longer lasting after-depolarization (apical, decay τ: 88.7 ± 17.7 ms, *n* = 9; basal, decay τ: 49.5 ± 4.2 ms, *n* = 22; *P* = 0.0039, unpaired *t*-test; Figure 2E). These data indicate that apical dendrites may have specific mechanisms for Ca^2+^-signal integration and modulation of dendritic excitability.

**Figure 3:**
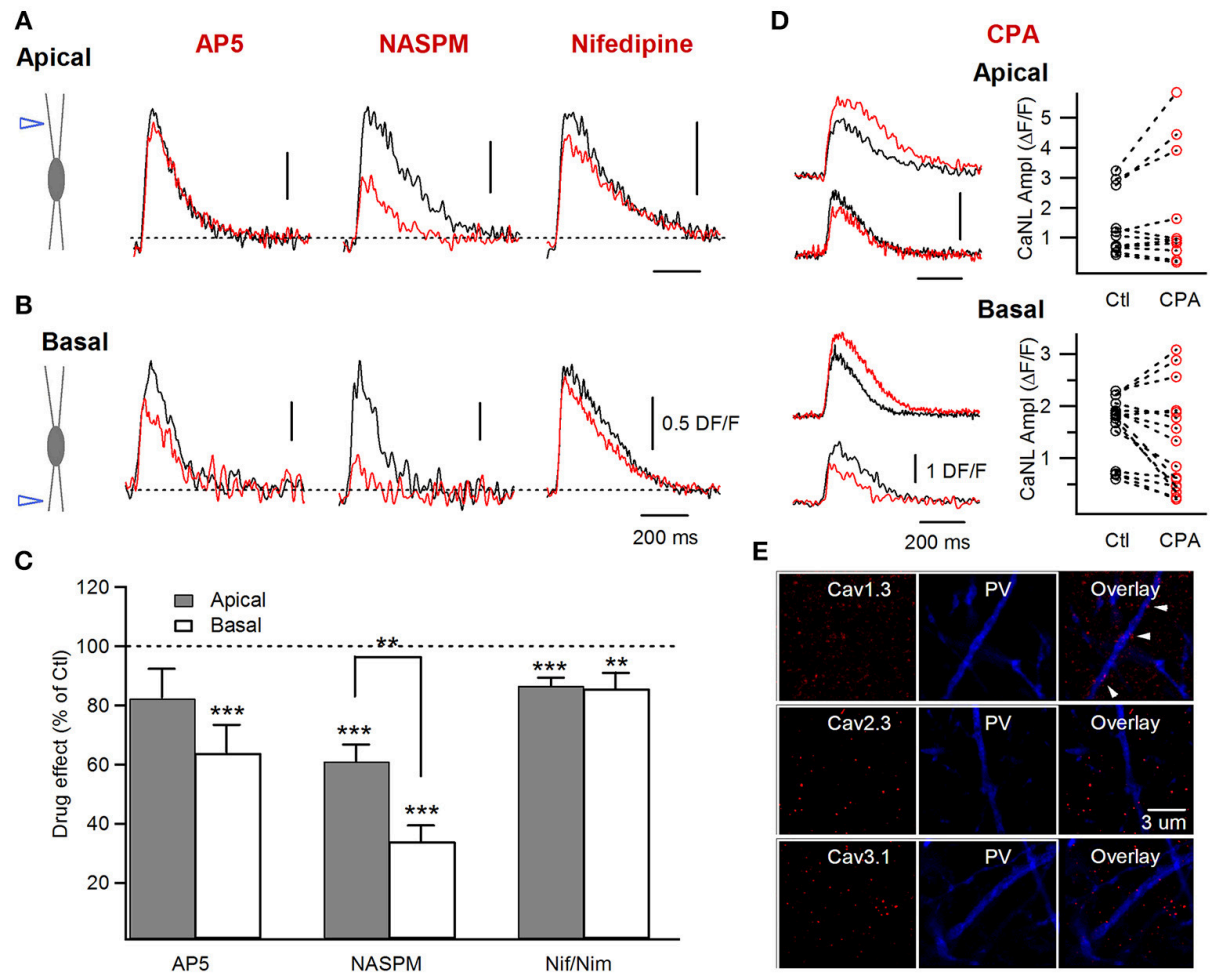
Postsynaptic Ca^2+^ sources involved in the generation of the supralinear Ca^2+^ events in apical vs. basal dendrites of FS cells. **(A,B)** Left, schematics of electrical stimulation in the area of apical (upper) or basal (lower) dendrites and examples of the supralinear Ca^2+^ transients recorded in control (black) and in the presence of AP5, NASPM and Nifedipine (red). **(C)** Summary bar graphs illustrating the drug effect (as a % of control) for a group of cells in the presence of different pharmacological agents in apical (gray) and basal (white) dendrites (AP5: apical *n* = 4, basal *n* = 8; NASPM: apical *n* = 5, basal *n* = 6; Nifedipine/Nimodipine: apical *n* = 7, basal *n* = 4). Note that the CP-AMPAR inhibitor NASPM had a major effect on the amplitude of the supralinear Ca^2+^ events in both dendritic domains. **(D)** Left, representative traces showing both an increase and a decrease in Ca^2+^ transient amplitude after application of SERCA pump blocker CPA in apical and basal dendrites. Right, graph showing the amplitude of Ca^2+^ transients in control and in the presence of CPA for individual dendritic segments (apical *n* = 5, 11 segments; basal *n* = 6, 16 segments). **(E)** Representative confocal images for Cav1.3, Cav2.3, Cav3.1 (left), and PV (middle) immunoreactivity and their overlay (right), illustrating the low expression of the Cav1.3 L-type VGCC subunit (white arrowheads) and the absence of expression for R-type Cav2.3 and T-type Cav3.1 VGCC subunits in apical dendrites of CA1 PV+ cells. **p < 0.01; ***p < 0.001.

To explore this possibility experimentally, we next compared the pharmacological profile of supralinear post-CaTs in different dendritic regions (Figure 3). As previous studies identified CP-AMPA receptors, NMDA receptors, L-type Ca^2+^-channels and intracellular Ca^2+^-release as four major sources of post-CaTs in FS interneurons (Goldberg et al., 2003a,b; Camiré and Topolnik, 2012, 2014; Chiovini et al., 2014; Hainmuller et al., 2014), we focused on these Ca^2+^-mechanisms by comparing their contribution to supralinear post-CaTs in different dendritic regions (Figures 3A–D). Our data showed that the supralinear post-CaTs recorded in apical dendrites were sensitive to CP-AMPA receptor inhibitor NASPM (CP-AMPAR-mediated Ca^2+^-response component: 39.0 ± 5.6%, *n* = 15 segments/5 cells; *P* = 0.0003; paired *t*-test; Figures 3A–C), although the CP-AMPAR component was significantly smaller in apical than in basal dendrites (*P* = 0.001; unpaired *t*-test; Figure 3C), pointing to the input-specific distribution of CP-AMPARs. Furthermore, the NMDAR contribution to supralinear Ca^2+^-responses in apical dendrites was not significant (NMDAR-mediated Ca^2+^-response component: 17.7 ± 9.5%, *n* = 13 segments/4 cells; *P* = 0.345; Wilcoxon signed-rank test; Figures 3A–C). We have noticed a high variability in the NMDAR component between individual dendritic segments in both dendritic regions (apical, range: 0–68%, *n* = 13 segments/4 cells; basal, range: 0–77%, *n* = 12 segments/8 cells), consistent with previous ultrastructural data on the highly variable NMDAR distribution in dendrites of PV+ cells (Nyíri et al., 2003). One bistratified cell showed an unexpected increase in basal post-CaT amplitude in the presence of AP5 via a yet unknown mechanism (Dore et al., 2017), and was not included in the current analysis.

In addition, the sensitivity of supralinear CaTs to the SERCA pump inhibitor CPA (30 μM) in apical dendrites was also highly variable. In 5 dendritic segments from 2 cells, application of CPA resulted in a significant decrease (to 66.2 ± 9.4% of control CaT; *P* = 0.043; Wilcoxon signed-rank test) of supralinear post-CaTs, pointing to the role of Ca^2+^-release in dendritic Ca^2+^ amplification (Figures 3A–C). However, three other cells (6 dendritic segments) showed an increase and prolongation of supralinear post-CaTs (to 142.6 ± 9.8% of control CaT; *P* = 0.028; Wilcoxon signed-rank test), consistent with the inhibition of ER uptake (Figure 5D). Taken together, these data indicate that intracellular Ca^2+^-stores, through microdomain-specific CICR or Ca^2+^ uptake, play an important role in supralinear Ca^2+^-dynamics across the dendritic tree of FS interneurons.

We also examined the contribution of the L-type voltage-gated Ca^2+^ channels (VGCCs) to the supralinear post-CaTs in apical vs. basal dendrites (Figures 3A–C). In line with previous findings on the role of this channel in the generation of dendritic Ca^2+^-spikes in apical dendrites of CA1 basket cells (Chiovini et al., 2014), our data showed that blocking L-type VGCCs with nifedipine (10 μM) or nimodipine (10 μM) had a variable, albeit statistically significant, effect on supralinear post-CaTs in apical dendrites of FS cells (L-type-Ca^2+^-response component, 13.4 ± 2.6%, *n* = 43 segments/6 cells, *P* = 0.000005; Wilcoxon signed-rank test; Figures 3A–C). The latter was likely due to the low channel density and variable distribution in PV+ dendrites, as revealed with immunolabeling for the Cav1.3 subunit (Figure 3E, upper). Also, we were unable to detect the R-type VGCC Cav2.3 (Figure 3E, middle) and the T-type VGCC Cav3.1 (Figure 3E, lower) subunits in PV+ apical dendrites, indicating that these types of VGCCs do not contribute to dendritic Ca^2+^-dynamics in FS cells. Taken together, the results of pharmacological experiments indicate that similar mechanisms shape postsynaptic Ca^2+^-signaling across the entire dendritic tree of FS cells, with a major role for CP-AMPARs in Ca^2+^-signal amplification, and highly variable and site-specific involvement of other mechanisms. Then, why do supralinear post-CaTs exhibit slower decay time in apical dendrites and why are they associated with a stronger somatic depolarization?

**Figure 4:**
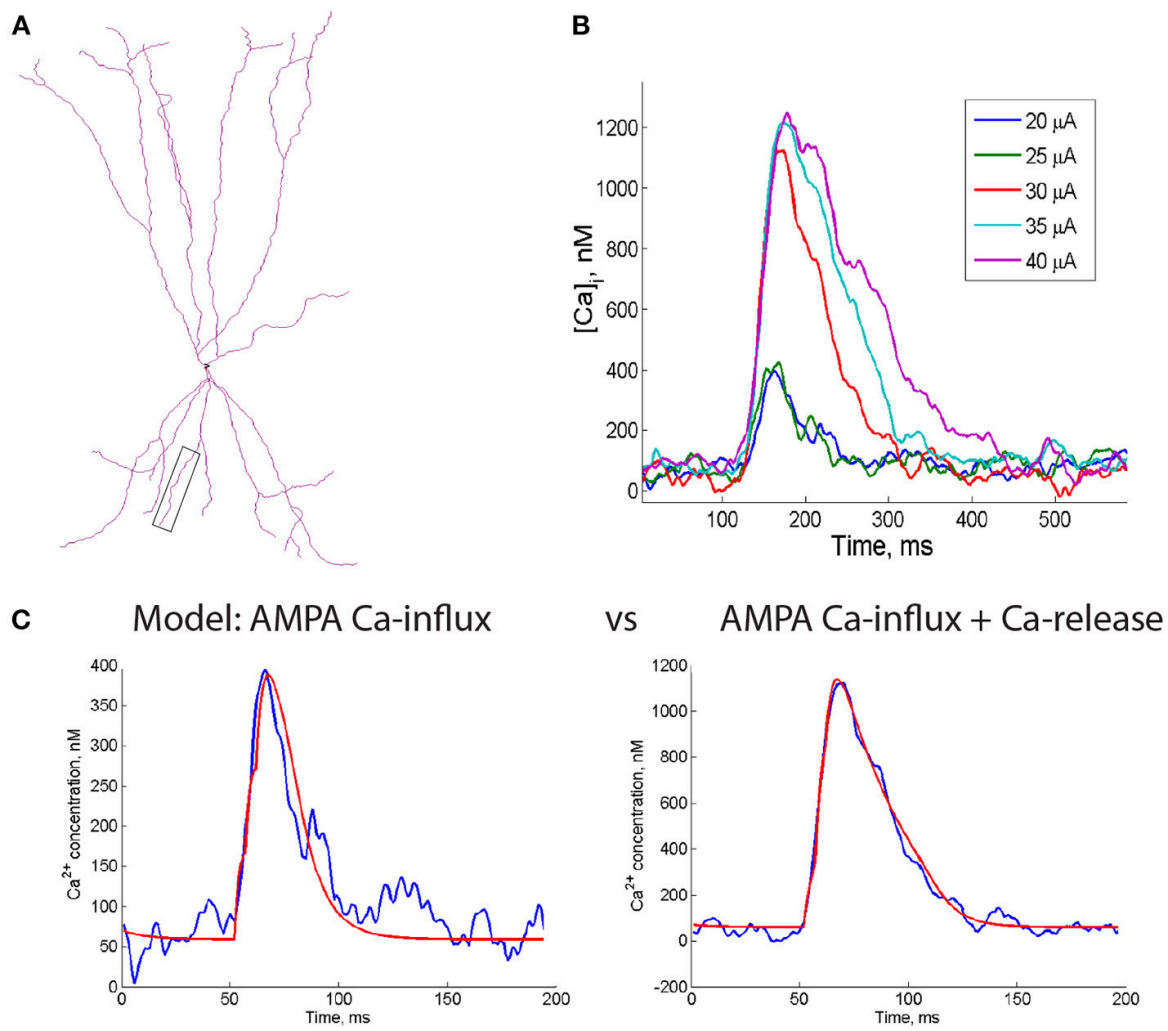
Computational model of the supralinear Ca^2+^ events in dendrites of FS interneurons. **(A)** Dendritic morphology of a reconstructed PV+ cell (Gulyás et al., 1999) that was used for simulations. Box indicates dendrite used for simulations in **(C)**. **(B)** The time course of changes in the postsynaptic Ca^2+^ concentration at different intensities of electrical stimulation (20 to 40 μA) calculated from the Ca^2+^ imaging experiment. Note a 3x rise in the peak post-CaT amplitude when the stimulus intensity was increased from 25 to 30 μA (by 20%), indicating the supralinear character of all following events. **(C)** Both subthreshold (left, blue trace) and supralinear (right, blue trace) Ca^2+^ events were accurately captured by the model (red fits), in which CP-AMPAR conductance was included alone (left) or together with the intracellular Ca^2+^ release (right). Note that no NMDA receptors or VGCCs were included in the model, indicating that these Ca^2+^ sources are dispensable for generation of the supralinear Ca^2+^ signals in FS cell dendrites.

Previous studies indicated that multiple factors can shape postsynaptic Ca^2+^ dynamics in neuronal dendrites, including the surface-to-volume ratio of dendritic compartments, the number of inputs activated synchronously during synaptic activity and the spatial distribution of Ca^2+^ sources and extrusion mechanisms (Topolnik et al., 2005; Losonczy and Magee, 2006; Scheuss et al., 2006; Branco and Häusser, 2011; Anwar et al., 2014; Bloss et al., 2018). To examine how these elements may impact the summation of post-CaTs in FS interneurons, we complemented experimental observations with a computational model of dendritic Ca^2+^-dynamics. We constructed a model based on realistic FS cell dendritic morphology (Figure 4A) with passive and active properties similar to those used previously for FS interneurons (Goldberg et al., 2003b; Nörenberg et al., 2010; Chiovini et al., 2014), which could reproduce experimental data on FS cell membrane properties, synaptic currents and Ca^2+^-dynamics (Camiré and Topolnik, 2014). To simulate minimally evoked and supralinear post-CaTs (Figure 4B), we used a simplified one-compartmental model, which is justified by the fact that, in FS interneurons, post-CaTs are generated in isolated dendritic microdomains (~5–10 μm) and, thus, weakly depend on somatic dynamics (Goldberg et al., 2003a; Camiré and Topolnik, 2014). Parameter optimization was performed to determine the values of biophysical parameters, which best fit the properties of the experimentally observed Ca^2+^-signals. We found that parameter values best fitting experimental recordings were in the following ranges: maximal RyR flux: 6.5 ± 0.5 ^*^ 10^−3^/ms, half-activation concentration *K*_*RyR*_: 0.9 ± 0.1 μM, Ca^2+^ decay time constant: 250 ± 60 ms, maximal rate and half-activation concentration of Ca^2+^ pumping from ER: 1 ± 0.3 ^*^ 10^−2^ μM/ms and 1.35 ± 0.2 μM, correspondingly. Both minimally evoked and supralinear post-CaTs were accurately captured by the model incorporating Ca^2+^-influx through CP-AMPA receptors and intracellular Ca^2+^-release with optimized parameters (Figure 4C).

**Figure 5:**
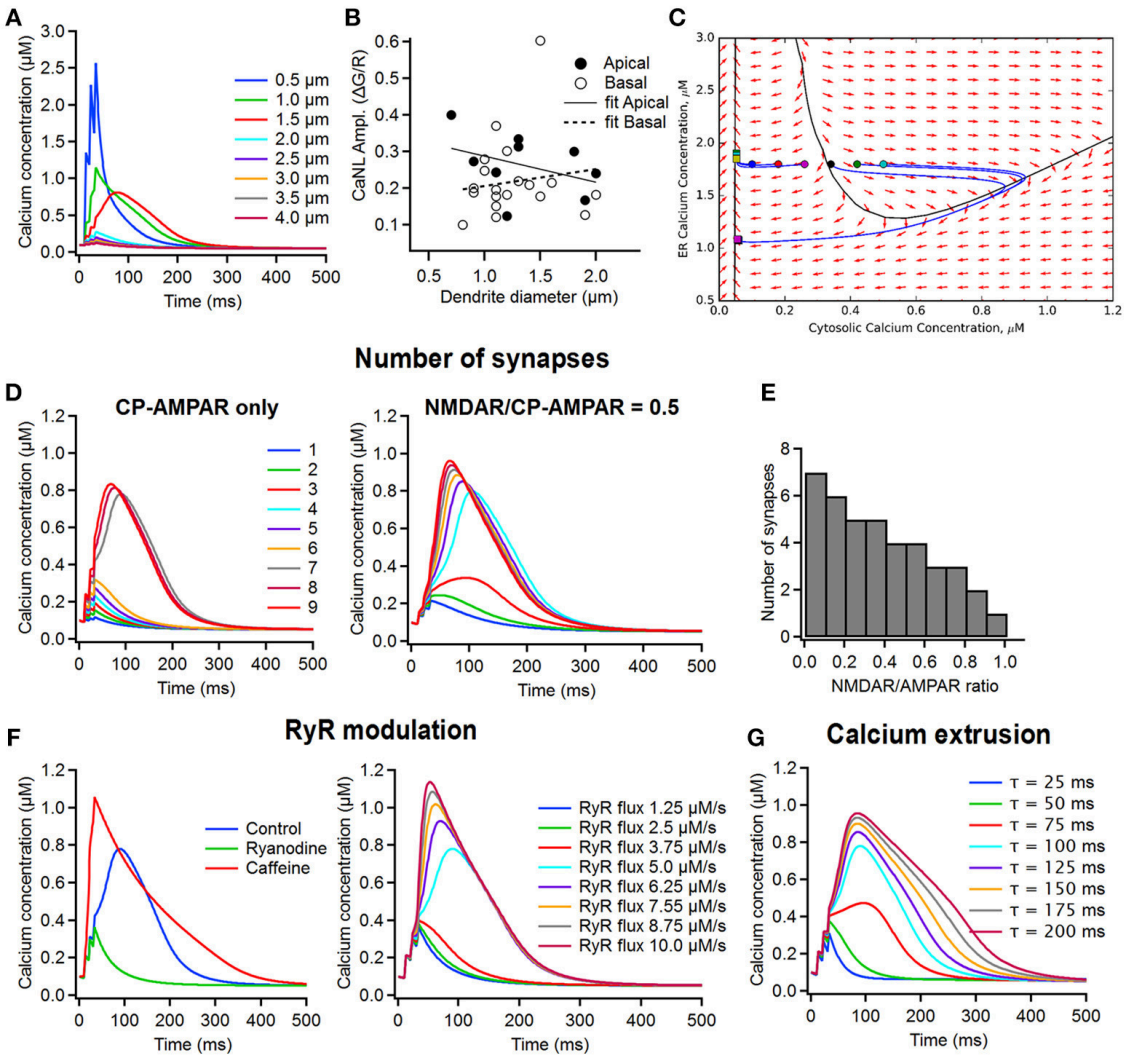
Computational simulations of the supralinear Ca^2+^ signals in dendrites of FS interneurons. **(A)** The time course of changes in the postsynaptic Ca^2+^ concentration in dendrites of different diameter (from 0.5 to 4 μm) in the model including only CP-AMPARs and CICR (left). Note that the thinner dendritic branches are predisposed to generating the supralinear Ca^2+^ events. **(B)** Summary plot showing the relationship between the CaT peak amplitude and the dendrite diameter in apical (closed symbols) and basal (open symbols) dendrites, with linear fits indicated by a continuous line for apical and dotted line for basal dendrites. No correlation was revealed between the amplitude of post-CaTs and the dendrite diameter. **(C)** Phase plane trajectory plots of the ER and cytosolic Ca^2+^ concentration. The black line represents the threshold for the generation of a CaNL, while the blue lines represents the course of changes in Ca^2+^ concentrations starting at different levels of cytosolic Ca^2+^ (circles) back to a baseline level (squares). **(D)** The time course of changes in Ca^2+^ concentration when a different number of synapses (from 1 to 9) was activated in the model with CP-AMPARs only (dendrite diameter, 1.5 μm) (left) and with a CP-AMPAR to NMDAR ratio of 0.5 (right). **(E)** The number of synapses required to trigger supralinear Ca^2+^ events depending on the AMPAR/NMDAR ratio. **(F)** The impact of RyR modulation on the time course of postsynaptic Ca^2+^ transients. Left, the effect of ryanodine (green) and caffeine on supralinear Ca^2+^ events; right, the time course of changes in Ca^2+^ concentration under different rates of RyR ion flux. Note that the amplitude of the supralinear Ca^2+^ event depends directly on the rate of the RyR ion influx. **(G)** The time course of changes in Ca^2+^ concentration depending on the speed of membrane Ca^2+^ removal via all Ca^2+^ extrusion mechanisms. Note that both the amplitude and the decay time constant of the supralinear Ca^2+^ event are modulated by the rate of Ca^2+^ extrusion.

First, we performed a set of simulation experiments that were structured to determine the impact of the surface-to-volume ratio on supralinear post-CaTs. When simulating post-CaTs in dendritic branches of different diameter (range, 0.5–4.0 μm), we found that generation of these signals is facilitated in thin dendrites (range, 0.5–1.5 μm; Figure 5A). However, no significant correlation between the diameter of dendritic branches in which Ca^2+^ imaging data were obtained and the amplitude of evoked CaTs was found in our experiments for both inputs (apical: 1.33 ± 0.14 μm; *n* = 10; *r* = −0.382; Pearson correlation; basal: 1.24 ± 0.07; *n* = 19; *r* = 0.081; Pearson correlation; Figure 5B).

Next, we estimated the number of synapses necessary for the generation of minimal and supralinear post-CaTs, and explored how it can affect the amplitude of these signals (Figures 5C,D). As the CP-AMPARs made a major contribution to supralinear post-CaTs (Figures 3A–C), at first, we considered the CP-AMPAR-containing synapses only. Using phase plane analysis, assuming that the cytosolic resting Ca^2+^ is fixed (70–100 nM; Aponte et al., 2008), we found that there exists a critical value for the resting endoplasmic reticulum (ER) Ca^2+^-level (ER-Ca^2+^) below which supralinear post-CaTs cannot be generated (Figure 5C). The amplitude of the Ca^2+^-event increased quasilinearly with the ER-Ca^2+^ concentration and the threshold for generation of these events stayed approximately constant in the concentration ranges of interest (threshold values equal to 372 ± 26 nM). For the optimal model parameter values, the system behaved linearly as soon as Ca^2+^ concentration stayed below the non-linearity threshold. The Ca^2+^ flux depends quasilinearly on the Ca^2+^ concentration, thus subthreshold Ca^2+^ dynamics may be approximated by a simple exponential relaxation process with a different effective Ca^2+^ decay time constant. The effective time constant found by least-squares fitting to Ca^2+^ flux vs. concentration dependence appeared to be in the range of 50 ± 15 ms. The linear relaxation approximation allowed analytical estimation of the minimal number of synapses activated to generate a supralinear post-CaT (Figure 5D). We found that, independent of the dendritic region, four synapses were activated during local synaptic stimulation to trigger a minimally evoked post-CaT observed typically in our experiments, and seven synapses were required to generate a supralinear signal (Figure 5D, left). Further increase in the number of synapses activated had no significant effect on the amplitude and decay time of post-CaT (Figure 5D, left), as the activation of RyRs always produced Ca^2+^-elevations of approximately the same amplitude and width. Collectively, these data indicate that differences in the decay time of supralinear post-CaTs observed are likely associated with site-specific distributions of kinetically slow Ca^2+^-sources and extrusion mechanisms.

As NMDARs could also make an important, albeit highly variable, contribution to supralinear post-CaTs, we also examined their impact on the number of synapses required for the generation of these signals (Figure 5E). In simulations where excitatory synapses with different CP-AMPA to NMDA receptor conductance ratio were activated, supralinear post-CaTs showed different threshold of induction and distinct decay kinetics (Figure 5E, left). In particular, increasing the NMDA receptor contribution resulted in larger amplitude and slower Ca^2+^-events that were now triggered by a lower number of synapses (Figure 5E, right). Therefore, including NMDA receptors in the model allowed us to decrease the minimal number of synapses necessary for the generation of supralinear Ca^2+^-elevations and increase their magnitude and duration, which could contribute to the experimentally observed high heterogeneity in Ca^2+^-signal gain across the dendritic tree.

In addition to the CP-AMPARs, the CICR via RyR activation represents a second important component of the supralinear post-CaTs in basal dendrites of FS cells (Camiré and Topolnik, 2014). To estimate whether this component may be responsible for the dendritic region-specific differences observed in the current study, we next explored the role of the RyR modulation on the magnitude of supralinear Ca^2+^-elevations. Consistent with experimental observations in basal dendrites of FS cells (Camiré and Topolnik, 2014), we found that supralinear post-CaTs were reduced to the minimally evoked Ca^2+^ level when simulating the effect of RYR antagonist ryanodine, and was potentiated in the model simulating the effect of caffeine (Figure 5F, left). In addition, varying the speed of Ca^2+^-flux through RyRs had a major impact on the amplitude and threshold of supralinear post-CaTs (Figure 5F, right), thus highlighting RyR modulation as a critical component in the Ca^2+^-signal summation. Next, given the important role of Ca^2+^ extrusion mechanisms in shaping dendritic Ca^2+^ signals (Scheuss et al., 2006), we also explored whether the slower supralinear post-CaTs in apical dendrites may result from different time constants of Ca^2+^ removal from the cytoplasm. By simulating different rates of activation of the Na^+^-Ca^2+^ exchangers (NCX) and membrane Ca^2+^-pumps (Figure 5G), we found that the activation of these mechanisms was efficient in controlling the width of supralinear post-CaTs, as well as the threshold for their induction and their amplitude (Figure 5G). Thus, our model predicts that the dendritic site-specific distribution and functional state of RyRs and Ca^2+^ extrusion mechanisms may be among the most important factors responsible for the observed difference in the decay time of supralinear post-CaTs and their overall variability.

## Discussion

Given the highly compartmentalized nature of dendritic signaling in different types of neurons (Goldberg et al., 2003a; Losonczy and Magee, 2006; Branco and Häusser, 2011), we combined experimental and computational studies in PV+ FS cells to examine the properties and the mechanisms of Ca^2+^ signals associated with activation of synaptic inputs in apical vs. basal dendrites. Using a combination of two-photon Ca^2+^ imaging and whole-cell patch-clamp recordings, we found significant differences in supralinear summation of Ca^2+^ signals, with a highly variable gain function, between the individual dendritic sites, further emphasizing the synapse-specific nature of dendritic Ca^2+^ integration in interneurons.

The CP-AMPARs made a major contribution to supralinear post-CaTs in all dendritic branches tested, with a stronger contribution in basal dendrites, pointing to the input-specific distribution of these receptors in FS cells. Compared with other types of cortical interneurons, FS PV+ cells exhibit the highest levels of GluA1, GluA3, and GluA4 mRNAs (Paul et al., 2017), pointing to the relative abundance of CP-AMPARs at their excitatory synapses. Furthermore, our data indicate that the CP-AMPAR–RyR interaction was critical for generation of supralinear post-CaTs in PV+ basket and bistratified cells, and in apical and basal dendrites that receive distinct excitatory inputs. These data indicate that all CP-AMPAR-containing synapses, regardless of the cell- and input-type, are capable of generating supralinear Ca^2+^ signals via AMPAR–RyR interaction. Interestingly, GluA1, 2, and 4 exhibit the sex-specific expression in dorsal hippocampus in response to early life experience, with a higher GluA1 and lower GluA2 and GluA4 content in females (Katsouli et al., 2014). These data indicate that CP-AMPAR Ca^2+^ signaling may dominate in females and underlie the sex-specific differences in learning and memory processes (Dachtler et al., 2011). Whether the sex-dependent GluA2 subunit expression affects hippocampal interneurons remains unknown but will be interesting to explore in relation to CP-AMPAR-CICR-mediated Ca^2+^ signaling and synaptic plasticity.

The NMDARs were also involved in the generation of supralinear post-CaTs in FS cells, albeit with a high degree of intercellular and intracellular variability. These data are consistent with the large variability in synaptic density of NR1, the obligatory NMDAR receptor subunit, in CA1 PV+ interneurons (Nyíri et al., 2003), and the cell type- as well as synapse-specific contribution of NMDAR subunits to glutamate transmission and long-term synaptic plasticity in interneurons (Lei and McBain, 2002; Lamsa et al., 2005, 2007; Galván et al., 2015; Akgül and McBain, 2016). Importantly, our data indicate that, at a subset of synapses, NMDARs are co-expressed with CP-AMPARs. Given the significant difference in activation kinetics between these two receptors, fast Ca^2+^ influx through CP-AMPARs precedes the activation of NMDARs and is involved in NMDAR inactivation (Rozov and Burnashev, 2016). It is therefore possible that at synapses with high CP-AMPAR/NMDAR ratio, NMDARs remain functionally silent, and a higher synaptic NMDAR fraction will be required for substantial contribution of these receptors to post-CaTs. It should be noted that the AMPAR/NMDAR contribution to postsynaptic Ca^2+^ influx may change depending on the level of synaptic activity, membrane polarization and state-dependent intracellular modulation and trafficking of different glutamate receptor subunits. These factors may all contribute to variability in contribution of CP-AMPARs and NMDARs to post-CaTs and may explain the fluctuation in the Ca^2+^ gain function.

To explore the biophysical basis of supralinear Ca^2+^ events, we constructed a computational model of FS basket cell. Analysis of simulations confirmed our experimental findings that supralinear Ca^2+^ signals in dendrites of FS cells rely mainly on Ca^2+^ influx at resting membrane potential through activation of CP-AMPARs with a subsequent Ca^2+^ release, and do not require the interaction with active conductances. This way the CP-AMPAR-enriched synapses can compensate for the relatively low densities of active ion conductances in dendrites of some interneurons, including PV+ basket and cerebellar stellate cells (Hu et al., 2014; Tran-Van-Minh et al., 2016). In this case, the CP-AMPAR–CICR-mediated amplification of local Ca^2+^ signals via generation of supralinear post-CaTs represents a simple voltage-independent way of synapse-specific modification of incoming inputs.

In addition, we examined the role of structural (dendrite diameter, number of clustered synapses) and functional (RyR and Ca^2+^ extrusion modulation) parameters in shaping the time course and magnitude of supralinear Ca^2+^ elevations. The model predicted that thin dendrites will be particularly prone to generation of supralinear post-CaTs, and that the number of the spatiotemporally clustered synapses required for such event to occur will depend on the CP-AMPAR/NMDAR ratio, with NMDARs decreasing the number of synapses necessary for supralinear Ca^2+^ integration. These data are in line with theoretical and experimental observations obtained in principal neurons, indicating that the synapse location, clustering, and composition are the leading factors in dendritic input integration (Rall, 1962; Holmes, 1989; Katz et al., 2009; Branco and Häusser, 2011; Anwar et al., 2014; Bloss et al., 2018). For simplicity, the CP-AMPAR-dependent inactivation of the NMDAR current (Rozov and Burnashev, 2016) was not included in the model but may have significant impact on the number of synapses required for generation of supralinear post-CaTs.

Our model also predicts that the properties and high variability of supralinear post-CaTs may depend on the functional state of the Ca^2+^ release, uptake and extrusion mechanisms, such as the RyR, Na^+^/Ca^2+^ exchanger and SERCA pump. RyRs are the largest ion channels known, which are able to generate significant Ca^2+^ flux from the ER to cytoplasm (Van Petegem, 2016; Williams et al., 2018). The 3 RyR isoforms (RyR1–RyR3) are expressed in the brain and are activated by cytosolic Ca^2+^, with RyR1 having a particularly high expression in the CA1 area (Hertle and Yeckel, 2007). Recent high-resolution imaging data revealed a tendency toward formation of irregular RyR clusters in cardiomyocytes (Macquaide et al., 2015). Whether the same spatial arrangement of RyRs may occur in central neurons remains unknown but could explain the fluctuating Ca^2+^ gain function during summation of post-CaTs along the dendritic tree. In addition, large amplitude CICR events occurring synchronously in several discrete loci were involved in membrane depolarization and generation of action potentials (Capogrossi et al., 1987). A similar mechanism could operate in apical dendrites of FS cells, where supralinear post-CaTs were associated with higher-level somatic depolarization and firing. Furthermore, recruitment of additional Ca^2+^ sources, such as mGluR-mediated mechanisms, may occur during specific patterns of activity and be coupled to activation of specific types of the transient receptor potential (Trp)-channels (Topolnik et al., 2005, 2006; Camiré and Topolnik, 2014; Hainmuller et al., 2014). Further studies will be required to understand their functional role in synapse- and cell type-specific amplification of interneuron dendritic Ca^2+^-signals.

The SERCA pump and NCX are considered the major mechanisms of cytosolic Ca^2+^ extrusion and complement each other in terms of Ca^2+^ affinities and transport capacity. They are also involved in control of pre- and postsynaptic Ca^2+^ dynamics, with direct impact on Ca^2+^-dependent signaling and synaptic plasticity (Blaustein and Lederer, 1999; Jeon et al., 2003; Scheuss et al., 2006; Empson et al., 2007). Modulating the time course of Ca^2+^ extrusion in the model had a significant impact on the duration and magnitude of supralinear post-CaTs, and also decreased the threshold for their induction. It is unknown whether the dendritic distribution of these mechanisms is uniform in these cells, but differences in Ca^2+^ extrusion could account for the slower decay of Ca^2+^ signals seen in apical dendrites experimentally. We found that this slower decay in turn lead to an increase in cumulative charge in apical dendrites. While we did not observe a difference in the induction of supralinear CaTs, the higher cumulative charge could facilitate Ca^2+^-dependent signaling in apical dendrites. These observations indicate that the dynamics of Ca^2+^ extrusion may be another important factor in the regulation of synaptic plasticity in interneurons.

Overall, the model reveals additional mechanisms important for supralinear integration of dendritic Ca^2+^ signals in FS cells. However, it does not explore all possible scenarios that can be activated during ongoing synaptic activity and coordinate dendritic Ca^2+^ dynamics and synaptic function. For example, we did not consider the role of dendritic GABAergic inhibition in regulation of Ca^2+^ signals, as our experiments were performed in the presence of GABA receptor blockers to isolate excitatory responses. Therefore, all GABAA receptor currents, including those involved in tonic inhibition, were abolished. Tonic inhibition is functional in some hippocampal interneuron populations (Song et al., 2011; Ferando and Mody, 2014; Pavlov et al., 2014). It is unclear whether it also operates in CA1 FS cells, but if this is the case, it may affect the spatial and temporal extent of supralinear Ca^2+^ events. Therefore, given the use of GABAA receptor blockers in our study to isolate excitatory responses, the physiological extend of our findings under normal conditions remains to be explored.

In addition, it has been suggested that mitochondria may be involved in CICR in certain conditions (Ichas et al., 1997). Moreover, the store-operated Ca^2+^ entry (SOCE), the mechanism through which the ER triggers Ca^2+^ influx following the store depletion, may contribute to Ca^2+^ signaling in neurons. While there is evidence that SOCE is involved in neuronal function (reviewed in Majewski and Kuznicki, 2015), the relatively low Ca^2+^ conductance of SOCE-associated channels makes them unlikely contributors to post-CaTs. However, following a massive release of Ca^2+^, the implication of these channels may become more important. Interestingly, SOCE is linked to L-type VGCC inhibition (Park et al., 2010). If this mechanism operates also in FS interneurons, it may explain the low contribution of L-type channels during Ca^2+^ non-linearities in our experiments.

More broadly, CP-AMPAR-mediated Ca^2+^ signaling has been implicated in the pathophysiology of several brain diseases. For example, the expression of CP-AMPARs increases following induction of experimental seizures (Rajasekaran et al., 2012; Lippman-Bell et al., 2016). In addition, CP-AMPAR-mediated Ca^2+^ signaling regulates depression-like behavior in chronic neuropathic pain (Goffer et al., 2013), and is among the first to respond to traumatic brain injury (Bell et al., 2007). It is clear that imbalanced CP-AMPAR Ca^2+^ signaling in dendrites of interneurons in combination with other factors may be involved in abnormal cellular and network computations. From this perspective, the identification of the CP-AMPAR interacting partners, and how these affect neuronal computations, will likely open new therapeutic avenues toward the prevention and treatment of several devastating disorders.

## Author Contributions

OC, TG, and LT performed experiments. IL performed computational simulations. OC and LT analyzed the data and wrote the manuscript.

### Conflict of Interest Statement

The authors declare that the research was conducted in the absence of any commercial or financial relationships that could be construed as a potential conflict of interest. The reviewer AS declared a shared affiliation, with no collaboration, with one of the authors, IL, to the handling editor at time of review.
